# Impact of Aspirin Use on Mortality and Cardiac Allograft Vasculopathy After Heart Transplantation: A Systematic Review and Meta‐analysis

**DOI:** 10.1111/ctr.70742

**Published:** 2026-11-07

**Authors:** Noémie Fournier, Panagiotis Antiochos, Baris Gencer, Anna Nowacka, Tamila Abdurashidova, Patrick Yerly, Philippe Meyer, Valentina Rancati, Zied Ltaief, Olivier Muller, Matthias Kirsch, Roger Hullin, Henri Lu

**Affiliations:** ^1^ Division of Cardiology Lausanne University Hospital (CHUV) University of Lausanne (UNIL) Lausanne Switzerland; ^2^ Division of Cardiac Surgery Lausanne University Hospital (CHUV) University of Lausanne (UNIL) Lausanne Switzerland; ^3^ Division of Cardiology Geneva University Hospitals (HUG) University of Geneva (UNIGE) Geneva Switzerland; ^4^ Division of Anesthesiology Lausanne University Hospital and University of Lausanne Lausanne Switzerland; ^5^ Department of Adult Intensive Care Medicine Lausanne University Hospital (CHUV) and University of Lausanne Lausanne Switzerland

**Keywords:** Aspirin, cardiac allograft vasculopathy, heart transplant, meta‐analysis

## Abstract

**Background:**

Cardiac allograft vasculopathy (CAV) remains a major cause of late graft failure and morbidity among heart transplant (HTx) recipients. Aspirin is currently assigned a Class IIb recommendation for CAV prevention, reflecting the limited evidence supporting its clinical benefit. We conducted a systematic review and meta‐analysis to assess the association between aspirin use and clinical outcomes in HTx recipients (PROSPERO ID: CRD420261399873).

**Methods:**

Outcomes of interest included all‐cause mortality and CAV‐related clinical outcomes (graft dysfunction or CAV‐related death). Literature searches were conducted through December 2025 in PubMed and EMBASE. Studies comparing HTx recipients treated with aspirin versus no aspirin were included. Hazard ratios (HRs) and 95% confidence intervals (CIs) were meta‐analyzed across studies.

**Results:**

Five observational studies were included, comprising four adult cohorts and one pediatric cohort. Participants were predominantly male (66.6%), with a mean age of ≈50 years in the four adult studies and 7.4 years in the sole pediatric study. Aspirin use was associated with significantly lower all‐cause mortality (four studies, HR 0.31 [95% CI: 0.15–0.67]). No statistically significant association was observed with CAV‐related clinical outcomes (HR 0.42 [95% CI: 0.10–1.77]).

**Conclusions:**

In HTx recipients, aspirin use was associated with lower all‐cause mortality, while no significant association was observed in the exploratory analysis of CAV‐related clinical outcomes. These findings should be interpreted cautiously given the observational design, substantial heterogeneity, and potential residual and time‐related biases. Prospective studies are needed to determine the efficacy and safety of aspirin after HTx.

## 1 Introduction

Heart transplantation (HTx) remains the gold‐standard treatment for end‐stage heart failure refractory to optimal medical therapy, with five‐year survival approaching 75% [1]. Despite these results, long‐term graft survival remains limited, largely due to cardiac allograft vasculopathy (CAV) [2].

CAV is the leading cause of late graft failure, mortality, and retransplantation after HTx, affecting nearly half of recipients within 10 years of transplantation and accounting for up to one third of deaths occurring between five and ten years post‐HTx [2]. Unlike conventional atherosclerosis, CAV is characterized by diffuse, concentric intimal hyperplasia resulting from a complex interplay of immune and non‐immune mechanisms, including platelet activation, endothelial injury, and smooth muscle cell proliferation [3, 4]. Its pathophysiology remains incompletely understood, and no curative treatment is available once the disease is established. Management therefore remains largely preventive [5, 6].

Current preventive strategies include optimization of immunosuppression, cardiovascular risk factor control, and cytomegalovirus prophylaxis [7]. Statin therapy carries a Class I recommendation in this setting [5, 6, 8]. Aspirin, through its antiplatelet and anti‐inflammatory properties, represents a biologically plausible preventive strategy; however, its use carries only a Class IIb recommendation in International Society for Heart and Lung Transplantation (ISHLT) guidelines, reflecting limited and inconsistent evidence [6]. To date, no randomized controlled trial has evaluated the effect of aspirin on CAV or post‐transplant mortality.

This systematic review and meta‐analysis aims to assess the effect of aspirin on all‐cause mortality and CAV‐related outcomes in HTx recipients, with the goal of informing clinical practice and guiding future prospective research.

## 2 Methods

### 2.1 Study Design and Protocol

This systematic review and meta‐analysis followed a prespecified study protocol, registered in the PROSPERO International Prospective Register of Systematic Reviews (ID: CRD420261399873). This work adhered to the recommendations of the International Committee of Medical Journal Editors [9], and followed the Preferred Reporting Items for Systematic Reviews and Meta‐Analyses (PRISMA) 2020 guidelines [10]. Ethical approval was not required, as our meta‐analysis was conducted at the study level using previously published data.

### 2.2 Literature Search Strategy and Data Collection

A systematic review of all relevant studies was performed, covering the period from inception to December 31, 2025, using the PubMed/MEDLINE (Medical Literature Analysis and Retrieval System Online) and EMBASE (Excerpta Medica Database) platforms. The following search terms were used: “aspirin”, “acetylsalicylic acid”, “antiplatelet”, “antiaggregant”, “cardiac allograft vasculopathy”, and “heart transplant.” All original research articles including HTx recipients, comparing aspirin users versus non‐users, were assessed for eligibility, without any restriction or filter on year of publication.

Studies were included in the analysis if they met the following criteria: original research articles published in English, comparison of aspirin users versus non‐users, availability of data on baseline population characteristics, documentation of at least one prespecified outcome, and reporting of hazard ratios (HRs) with 95% confidence intervals (CIs) for at least one outcome. Patients of any age, sex, and indication for HTx were included. Clinical case reports, review articles, meta‐analyses, conference abstracts, and non‐human research were excluded.

Two authors (N.F. and P.A.) independently assessed study eligibility; reference management was performed using the Covidence software. Any discrepancy was resolved through consensus. Data extraction was independently performed by two authors (N.F. and P.A.), and any disagreement was adjudicated by a third author (H.L.). The quality of each included study was independently evaluated by two authors (N.F. and A.N.) using the Newcastle−Ottawa Scale [11]. In addition, for each study, we extracted the definition and timing of aspirin exposure, the analytic time origin, and the handling of deaths or clinical events occurring before aspirin exposure could be classified.

### 2.3 Outcomes

The primary outcome was all‐cause mortality. The secondary analysis evaluated CAV‐related clinical outcomes, including CAV‐related graft dysfunction and CAV‐related death, and was considered exploratory.

### 2.4 Statistical Analyses

For each study, baseline patient characteristics were extracted, with continuous variables summarized as means ± standard deviations (SDs) or medians (interquartile ranges [IQRs]), and categorical variables reported as counts with percentages.

A random‐effects model was used to account for population diversity and methodological variation among studies, and heterogeneity across studies was assessed using the Cochran Q statistic and the Higgins and Thompson heterogeneity test (I^2^). The I^2^ statistic was used to quantify the proportion of variation across studies attributable to heterogeneity rather than chance, with thresholds defined as follows: less than 25% indicating low heterogeneity, 25%–50% moderate heterogeneity, and greater than 50% high heterogeneity. HRs and their corresponding 95% CIs were extracted from each study and log‐transformed prior to inclusion in the meta‐analysis. When available, estimates adjusted for baseline characteristics were preferentially used. When an HR was not directly reported, individual time‐to‐event data were reconstructed, whenever possible, from the published Kaplan–Meier curve and numbers at risk using the iterative algorithm described by Guyot et al. [12]. The corresponding HR and 95% CI were then estimated using a Cox proportional hazards model.

The meta‐analysis was conducted on the natural log‐transformed HR scale; pooled estimates were subsequently exponentiated and presented on the original HR scale. All p‐values were two‐sided, and values less than 0.05 were considered statistically significant. Statistical analyses were performed using Stata (version 19; StataCorp LLC, College Station, TX).

## 3 Results

### 3.1 Study Selection

A total of 3,010 references were initially identified. After removal of duplicates and screening of titles and abstracts, 14 references were selected for full‐text review. Of these, 9 were excluded for the following reasons: 5 were conference abstracts, 2 had a wrong study design, 1 was a meta‐analysis, and 1 had missing outcome data. Five studies met the inclusion criteria and were included in the systematic review (Figure 1) [13, 14, 15, 16, 17]. Methodological quality, assessed using the Newcastle–Ottawa Scale, was moderate or high across all included studies (Table S1).

**FIGURE 1:**
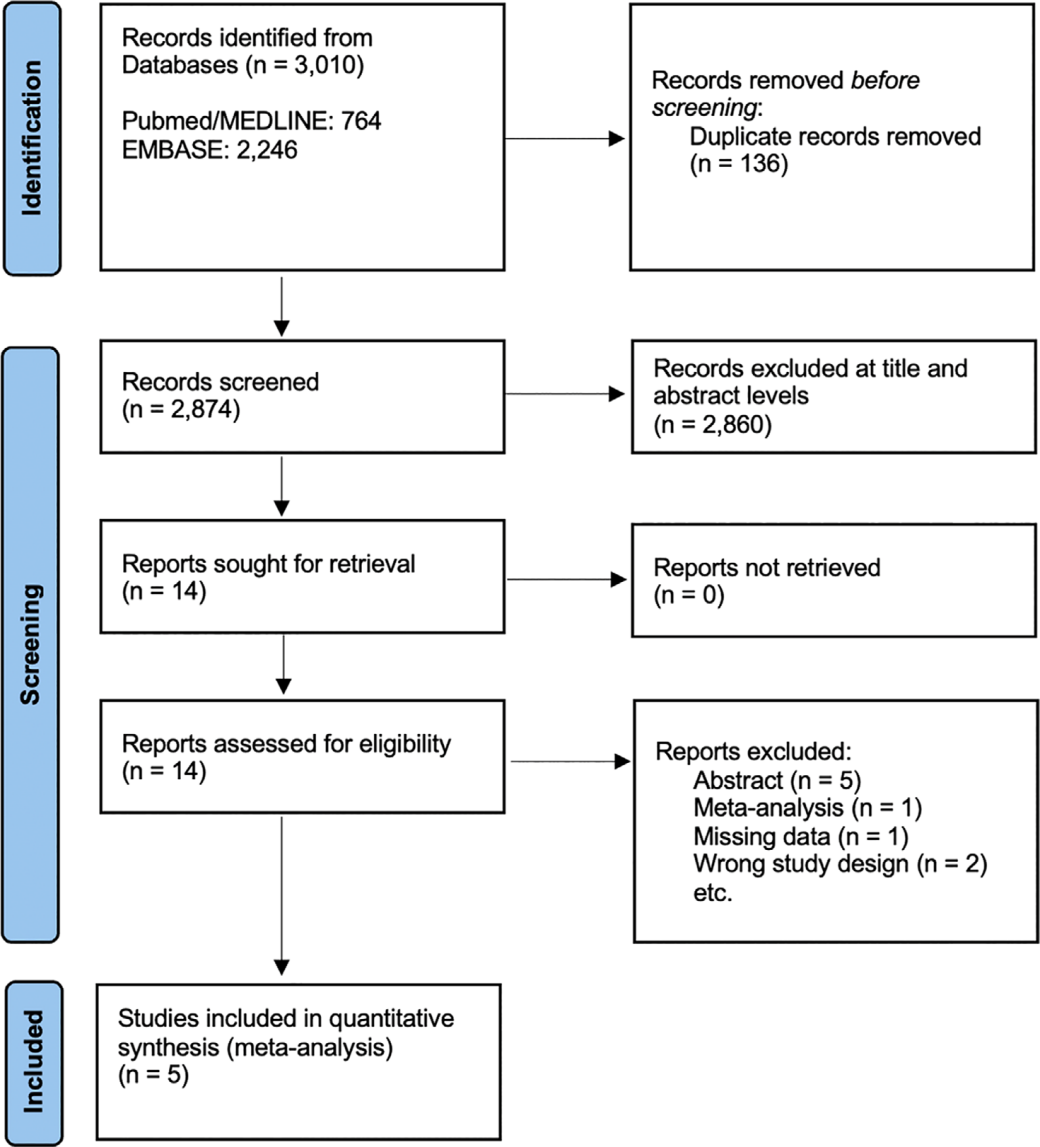
PRISMA Flow Diagram. Abbreviations: EMBASE, Excerpta Medica Database; MEDLINE, Medical Literature Analysis and Retrieval System Online; PRISMA, Preferred Reporting Items for Systematic Reviews and Meta‐Analyses.

### 3.2 Definitions of Outcomes and Aspirin Exposure

All‐cause mortality was reported in four of the five included studies [13, 14, 15, 17]. The only study that did not report this outcome was D'Addese et al., which was also the only exclusively pediatric study [16].

CAV‐related outcomes varied across studies. Asleh et al. reported CAV‐related graft dysfunction, defined as allograft dysfunction associated with known CAV in the absence of significant rejection or significant organic tricuspid valve regurgitation [14]. Kim et al. reported CAV‐related death, in addition to angiographic CAV of varying severity [13]. D'Addese et al. assessed incident CAV after a 3‐year CAV‐free landmark [16]. Peled et al. assessed first occurrence of CAV and a combined endpoint of CAV or all‐cause mortality [15], whereas Kessler Iglesias et al. assessed moderate‐to‐severe CAV (ISHLT grade ≥2) [17]. CAV was assessed by coronary angiography in all studies according to the 2010 ISHLT nomenclature [18]. Serial three‐dimensional intravascular ultrasound (IVUS) was additionally performed systematically in Asleh et al. and at the discretion of individual centers in D'Addese et al. [14, 16]. Study‐specific CAV‐related outcome definitions are summarized in Table 1.

Definitions and timing of aspirin exposure varied across studies (Table 1). Asleh et al. defined early aspirin use as administration within the first post‐transplant year; time‐to‐event analyses were anchored at the 1‐year assessment, only recipients with 1‐year follow‐up and CAV assessment were included, and aspirin exposure was additionally modeled as a time‐dependent covariate [14]. Kim et al. classified patients as aspirin users if aspirin was prescribed for at least 6 months during the first post‐transplant year and excluded patients who died during that first year [13]. Kessler Iglesias et al. classified exposure according to aspirin commencement during the index transplant hospitalization, with patients dying before discharge excluded [17]. Peled et al. defined early aspirin use as initiation within the first post‐transplant month and excluded patients who died during that month [15]. D'Addese et al. classified aspirin exposure over the first 3 post‐transplant years; eligibility required survival for at least 3 years without CAV, and the 3‐year follow‐up visit served as the analytic landmark [16]. Given differences in population, study design, and outcome definition, the pediatric study was retained in the systematic review but was not included in the quantitative CAV‐related analysis [16].

### 3.3 Baseline Characteristics

A total of 1,983 patients were included, with 730 (36.8%) in the aspirin group and 1,253 (63.2%) in the no‐aspirin group (Table 2). The study population was predominantly male (66.6%) with a mean age of approximately 50 years across the four adult studies [13, 14, 15, 17]. The study by D'Addese et al. included an exclusively pediatric population, with a mean age of 7.3 ± 5.9 years in the aspirin group and 7.4 ± 6.1 years in the no‐aspirin group [16]. The mean follow‐up duration across studies was 7.1 years (range: 3–15 years).

**TABLE 1:** Study‐level definitions of aspirin exposure, handling of early events, and CAV‐related outcomes.

Study	Aspirin exposure definition	Handling of deaths/events before exposure classification / analytic time origin	CAV‐related outcome(s) reported
Asleh et al. [14]	Early ASA within first post‐transplant year; late ASA thereafter	Only recipients with 1‐year follow‐up and CAV assessment included; follow‐up for clinical events from year 1; ASA additionally modeled as a time‐dependent covariate	CAV‐related graft dysfunction; CAV‐related death; nonfatal and overall CAV‐related events; IVUS progression
D'Addese et al. [16]	ASA during first 3 post‐transplant years; continuous/intermittent/none	Required survival ≥3 years without CAV; deaths and CAV before 3 years excluded; 3‐year visit used as landmark	Incident CAV after 3‐year CAV‐free landmark
Kim et al. [13]	ASA for ≥6 months during first post‐transplant year	Patients dying during first post‐transplant year excluded	CAV grade ≥2; any CAV; grade 3 CAV; CAV‐related death
Kessler Iglesias et al. [17]	ASA commenced during index transplant hospitalization	Patients dying before discharge excluded	Moderate/severe CAV (ISHLT grade ≥2)
Peled et al. [15]	ASA initiated within first post‐transplant month	Patients dying within first month excluded	First occurrence of CAV; CAV or all‐cause mortality

Abbreviations: ASA, acetylsalicylic acid; CAV, cardiac allograft vasculopathy; ISHLT, International Society for Heart and Lung Transplantation; IVUS, intravascular ultrasound.

**TABLE 2:** Baseline patient characteristics.

Study	Arm	N	Age (years)	Male (%)	HTN (%)	DM (%)	Smoking (%)	Dyslipidemia (%)	Statin use (%)	CMV (%)	CCB (%)
Asleh et al. [14]	*No‐ASA*	455	49.4 ± 13.7	67.5	40.9	20.9	**—**	**—**	82.9	**—**	50.8
—	*ASA*	74	53.1 ± 12.6	71.6	41.9	24.3	**—**	**—**	87.8	—	45.9
D'Addese et al. [16]	*No‐ASA*	378^*^	7.4 ± 6.1	56.6	**—**	**—**	**—**	**—**	42.1	—	—
—	*ASA*	378^*^	7.3 ± 5.9	55.0	**—**	**—**	**—**	**—**	41.0	—	—
Kim et al. [13]	*No‐ASA*	61	55.0 (44.1, 63.1)	59.0	82.0	13.1	**—**	2.5 (2.0, 3.3) (LDL‐C levels, mmol/L)	73.8	29.5	73.8
—	*ASA*	59	54.9 (47.3, 62.2)	89.8	89.8	28.8	**—**	2.6 (2.2, 3.2) (LDL‐C levels, mmol/L)	79.7	18.6	74.6
Kessler Iglesias et al. [17]	*No‐ASA*	250	49.8	75.6	28.2	14.9	23.8	19.0	87.6	**—**	27.2
—	*ASA*	122	49.1	74.6	14.8	15.6	41.0	9.5	88.5	**—**	15.6
Peled et al. [15]	*No‐ASA*	109	45 ± 17	86.0	34.0	20.0	40.0	49.0	91.0	25.3	—
—	*ASA*	97	52 ± 11	77.0	38.0	20.0	40.0	46.0	94.0	14.0	—

*Note:* Continuous variables are expressed as means ± standard deviations, or medians (interquartile range).

Abbreviations: ASA, acetylsalicylic acid; CCB, calcium channel blocker; CMV, cytomegalovirus; DM, diabetes mellitus; HTN, hypertension; LDL‐C, low‐density lipoprotein cholesterol. Values are reported as mean ± standard deviation or percentage.—indicates data not reported.

^*^Propensity‐matched cohort.

Statin therapy was prescribed in the vast majority of patients across all adult studies, ranging from 73.8% to 94.0% overall, with no statistically significant difference between groups in most studies. Renal function and lipid profile were similarly distributed between groups across studies reporting these variables.

Regarding comorbidity distribution, patients receiving aspirin had a significantly higher prevalence of active smoking in the study by Kessler Iglesias et al. (41.0% vs. 23.8%; *p* = 0.004) [17] and were older in the studies by Asleh et al. (53.1 ± 12.6 vs. 49.4 ± 13.7 years; *p* = 0.032) and Peled et al. (52 ± 11 vs. 45 ± 17 years; *p* < 0.001) [14, 15]. Cytomegalovirus infection was less frequently reported in aspirin‐treated patients in two studies [13, 15]. No statistically significant differences between groups were observed for diabetes, hypertension, or lipid profile in most included studies.

### 3.4 Aspirin Use and Outcomes

Based on the results of four studies [13, 14, 15, 17], aspirin use was associated with a significantly lower risk of all‐cause mortality (pooled HR 0.31; 95% CI 0.15–0.67; *p* for heterogeneity = 0.01; Figure 2). The degree of heterogeneity was high (I^2^ = 72.8%). For Peled et al., the all‐cause mortality HR was derived from reconstruction of the published Kaplan–Meier curve for death alone [15].

**FIGURE 2:**
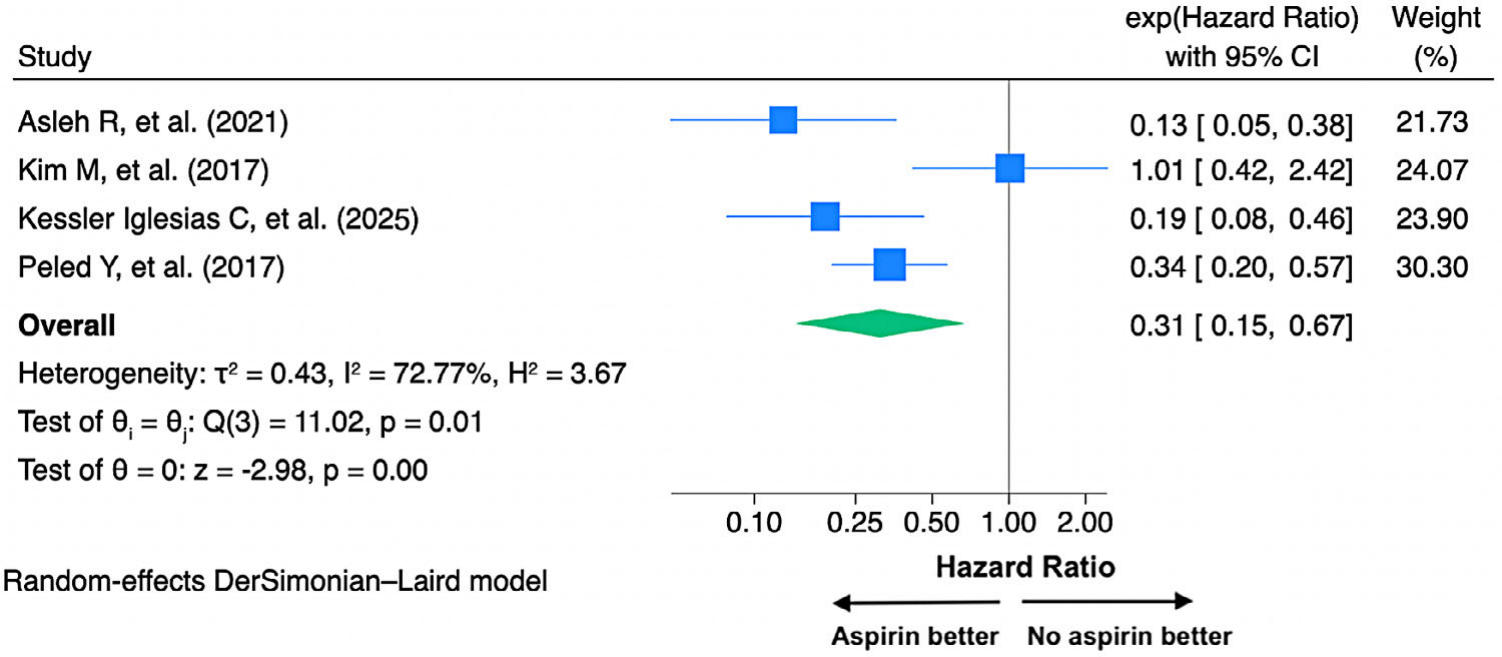
Association of Aspirin Use with All‐Cause Mortality. For Peled et al., the hazard ratio for all‐cause mortality was estimated from reconstructed individual time‐to‐event data from the published Kaplan–Meier curve [15]. CI, confidence interval; HR, hazard ratio.

Two adult studies contributed to the exploratory analysis of CAV‐related clinical outcomes: Asleh et al. reported CAV‐related graft dysfunction, whereas Kim et al. reported CAV‐related death [13, 14]. Aspirin use was not significantly associated with these outcomes (HR 0.42, 95% CI 0.10–1.77; I^2^ = 51.1%; *p* for heterogeneity = 0.15; Figure 3).

**FIGURE 3:**
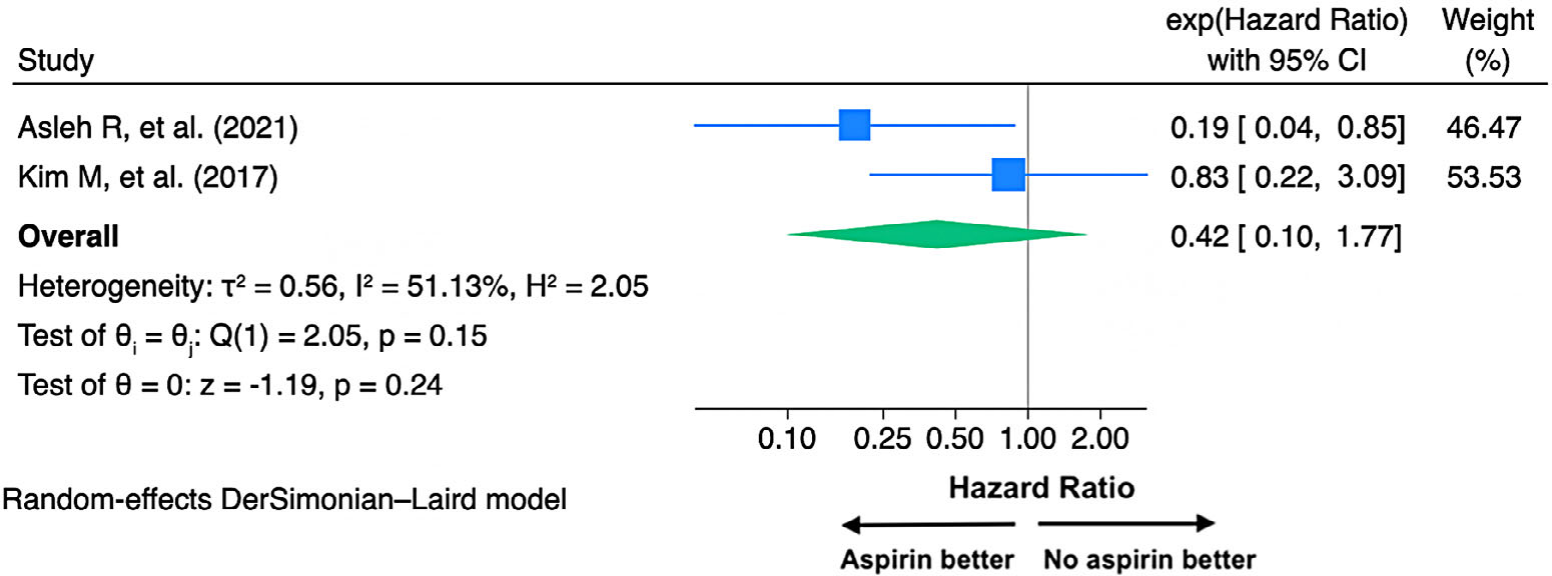
Association of Aspirin Use with CAV‐Related Graft Dysfunction or CAV‐Related Death in Heart Transplant Recipients. CAV, cardiac allograft vasculopathy; CI, confidence interval; HR, hazard ratio.

## 4 Discussion

In this meta‐analysis of 1,983 HTx recipients across five retrospective studies, aspirin use was associated with lower all‐cause mortality, although substantial between‐study heterogeneity was present. No statistically significant association was observed in the exploratory analysis of CAV‐related clinical outcomes.

These findings extend and update a prior systematic review, which included seven observational studies encompassing all antiplatelet agents and searched through April 2020 [19]. That review differed from the present work in several respects: it included conference abstracts, and only two of its seven studies reported all‐cause mortality, yielding a neutral pooled estimate (HR 0.95; 95% CI 0.67–1.34). By pooling four mortality‐reporting studies, the present analysis achieves substantially greater statistical power and yields a markedly different conclusion, suggesting a potential survival benefit.

The magnitude of the observed mortality association must nonetheless be interpreted in the context of important heterogeneity across studies. Such a large effect size is unusual in observational cardiovascular research and possibly reflects, at least in part, residual confounding inherent to the non‐randomized design of the included studies. Patients receiving aspirin may have differed systematically from those not receiving aspirin, including with respect to bleeding risk, renal dysfunction, frailty, or malignancy, all of which may influence both treatment selection and prognosis. In addition, the definition and timing of aspirin initiation differed considerably. Asleh et al. showed that early aspirin use—within the first post‐transplant year—was associated with an 87% mortality reduction and significant attenuation of CAV progression on serial IVUS, while late initiation was paradoxically associated with worse plaque progression, possibly reflecting confounding by indication [14]. Kessler Iglesias et al. and Peled et al. reported mortality risk reductions of 81% and 66 =% respectively, suggesting early antiplatelet protection [15, 17]. By contrast, Kim et al.—who defined aspirin exposure as at least six months of use during the first post‐transplant year without specifying exact timing—observed no significant mortality benefit, possibly reflecting dilution of the treatment effect by later initiators within that exposure definition [13].

Potential time‐related bias warrants careful consideration given the magnitude of the observed mortality association. Importantly, deaths occurring before fulfillment of the aspirin exposure definition were generally not assigned to the non‐aspirin group. Asleh et al. anchored clinical‐event analyses at the 1‐year post‐transplant assessment and additionally modeled aspirin exposure as a time‐dependent covariate, whereas Kim et al. excluded recipients who died during the first post‐transplant year and Peled et al. excluded those who died within the first post‐transplant month [13, 14, 15]. D'Addese et al., which did not contribute to the mortality meta‐analysis, used an explicit 3‐year CAV‐free landmark [16]. These approaches reduce the potential for classic immortal‐time misclassification of early deaths. However, conditioning study inclusion or analytic follow‐up on survival through the exposure‐assessment period may introduce survivor‐selection bias and limits inference regarding the earliest post‐transplant period. Because individual participant data were unavailable, the magnitude of any residual time‐related or survivor‐selection bias could not be quantified.

The exploratory CAV‐related analysis included only two adult studies reporting CAV‐related graft dysfunction or CAV‐related death, resulting in limited precision of the pooled estimate [13, 14]. D'Addese et al. was retained in the systematic review but was not quantitatively pooled because it assessed incident CAV after a 3‐year CAV‐free landmark [16].

The biological rationale for these observations is well established. HTx recipients exhibit enhanced platelet reactivity, driven in part by calcineurin inhibitor therapy, as well as endothelial injury triggered by ischemia‐reperfusion, allograft rejection, and cytomegalovirus infection—all of which are recognized contributors to CAV pathogenesis [2, 3, 4]. Activated platelets promote monocyte recruitment and smooth muscle cell proliferation, ultimately leading to the diffuse concentric intimal hyperplasia that characterizes CAV [20]. Through irreversible cyclooxygenase‐1 inhibition and suppression of thromboxane A_2_‐mediated platelet activation, aspirin may attenuate this early thrombo‐inflammatory component of CAV development [21]. Beyond its antiplatelet effects, aspirin may also attenuate the local inflammatory response through inhibition of prostaglandin synthesis, potentially limiting endothelial activation and leukocyte recruitment at sites of early vascular injury [21]. Aspirin may confer particular benefit in recipients with prior allograft rejection, a group in whom thrombo‐inflammatory injury is especially pronounced, with an observed 83% reduction in the risk of CAV grade ≥2 in this subgroup [22]. However, platelet aggregation has been shown to remain significantly elevated in some transplant recipients with established CAV despite aspirin therapy, raising the possibility of clinically meaningful aspirin resistance in this population, and suggesting that more potent antiplatelet strategies may ultimately be required [23].

While aligned with the current Class IIb recommendation in ISHLT guidelines [6], the magnitude of the observed mortality signal, alongside the biological plausibility of aspirin's antiplatelet and anti‐inflammatory effects in this setting, support consideration of early aspirin initiation after HTx in appropriately selected recipients without contraindications. These findings should nonetheless be interpreted with caution given the risk of immortal time bias and confounding by indication inherent to observational data. A prospective randomized controlled trial—designed specifically to evaluate aspirin initiated within days of transplantation—remains the essential next step to define the efficacy, safety, and optimal use of antiplatelet therapy in this setting.

### 4.1 Limitations

Our study has several potential limitations. First, the absence of randomized controlled trials comparing aspirin use versus non‐use in HTx recipients limits this meta‐analysis to observational studies, which are inherently subject to confounding and selection bias. In particular, definitions and timing of aspirin exposure differed substantially across studies. Although deaths occurring before exposure ascertainment were generally excluded rather than assigned to the non‐aspirin group, several studies conditioned eligibility or analytic follow‐up on survival through the exposure‐assessment period, introducing potential survivor‐selection bias. Second, CAV was assessed using variable imaging modalities across studies (ranging from coronary angiography alone to serial three‐dimensional IVUS), resulting in heterogeneity in diagnostic sensitivity that may have influenced outcome ascertainment. Third, concomitant medications, including immunosuppressive regimens and statin therapy, varied considerably across studies and time periods, representing potential sources of residual confounding. Finally, none of the included studies reported systematic data on bleeding events associated with aspirin use, precluding any assessment of the benefit‐risk balance at the individual patient level.

## 5 Conclusions

In this meta‐analysis of observational studies, early aspirin use after HTx was associated with lower all‐cause mortality. No significant association was observed in the exploratory analysis of CAV‐related clinical outcomes. Given substantial heterogeneity, potential residual and time‐related biases, and limited safety data, these findings should be considered hypothesis‐generating. A randomized controlled trial evaluating aspirin initiated early after HTx remains the essential next step to confirm efficacy, define optimal timing, and quantify bleeding risk.

## Funding

The authors have nothing to report.

## Conflicts of Interest

The authors declare no conflicts of interest.

## Supporting information




**Supporting Information**: ctr70742‐sup‐0001‐SuppMat.docx

## Data Availability

All data underlying this systematic review and meta‐analysis were obtained from previously published studies and are available in the original articles. The data extracted and analyzed for this study are available from the corresponding author upon reasonable request.
