# Supplementary material for: Impact of Aspirin Use on Mortality and Cardiac Allograft Vasculopathy After Heart Transplantation: A Systematic Review and Meta‐analysis
Source: Clin Transplant. 2026 Nov 7;40(11):e70742. doi: 10.1111/ctr.70742 (PMC13744394; doi:10.1111/ctr.70742)
Supplement: Supplementary file 1 [file CTR-40-e70742-s001.docx]

**Supplementary data**

**Impact of Aspirin Use on Mortality and Cardiac Allograft Vasculopathy After Heart Transplantation: A Systematic Review and Meta-analysis**

Noémie Fournier, Panagiotis Antiochos, Baris Gencer, Anna Nowacka, Tamila Adburashidova, Patrick Yerly, Philippe Meyer, Valentina Rancati, Zied Ltaief, Olivier Muller, Matthias Kirsch, Roger Hullin, Henri Lu

**Supplementary Table 1.** Methodological Quality Assessment of Included Studies Using the Newcastle–Ottawa Scale

**Supplementary Table 1.** Methodological Quality Assessment of Included Studies Using the Newcastle–Ottawa Scale

| **Study** | **Study Design** | **SELECTION** | | | | **COMPARABILITY** | **OUTCOMES** | | | **Total Score** |
| --- | --- | --- | --- | --- | --- | --- | --- | --- | --- | --- |
|  |  | **Representativeness of exposed cohort** | **Selection of non-exposed cohort** | **Ascertainment of exposure** | **Outcome not present at start of study** | **Comparability of cohorts (design or analysis)** | **Assessment of outcomes** | **Length of follow-up** | **Adequacy of follow-up** |  |
| **Asleh R, et al. (2021)** | *Retrospective* | **★** | **★** | **★** | **★** | **★** | **★** | **★** | — | **7/9** |
| **D'Addese L, et al. (2023)** | *Retrospective* | **★** | **★** | **★** | **★** | **★** | **★** | **★** | — | **7/9** |
| **Kim M, et al. (2017)** | *Retrospective* | **★** | **★** | **★** | **★** | **★★** | **★** | **★** | — | **8/9** |
| **Kessler Iglesias C, et al. (2025)** | *Retrospective* | **★** | **★** | **★** | **★** | **★★** | **★** | **★** | **★** | **9/9** |
| **Peled Y, et al. (2017)** | *Retrospective* | **★** | **★** | **★** | **★** | **★★** | **★** | **★** | — | **8/9** |

Each domain is scored with one star (★) except Comparability, which allows up to two stars (★★). Maximum total score: 9. — indicates criterion not met or not applicable.
